# PR3 and Elastase Alter PAR1 Signaling and Trigger vWF Release via a Calcium-Independent Mechanism from Glomerular Endothelial Cells

**DOI:** 10.1371/journal.pone.0043916

**Published:** 2012-08-29

**Authors:** Samantha P. Tull, Anne Bevins, Sahithi Jyothsna Kuravi, Simon C. Satchell, Bahjat Al-Ani, Stephen P. Young, Lorraine Harper, Julie M. Williams, George Ed Rainger, Caroline O. S. Savage

**Affiliations:** 1 Schools of Immunity and Infection, College of Medicine and Dentistry, University of Birmingham, Birmingham, United Kingdom; 2 Clinical and Experimental Medicine, College of Medicine and Dentistry, University of Birmingham, Birmingham, United Kingdom; 3 WTCRF, Queens Elizabeth Hospital, Birmingham, Academic Renal Unit, University of Bristol, Bristol, United Kingdom; 4 Academic Renal Unit, University of Bristol, Bristol, United Kingdom; 5 Department of Physiology, College of Medicine, King Khalid University, Abha, Saudi Arabia; Institut National de la Santé et de la Recherche Médicale, France

## Abstract

Neutrophil proteases, proteinase-3 (PR3) and elastase play key roles in glomerular endothelial cell (GEC) injury during glomerulonephritis. Endothelial protease-activated receptors (PARs) are potential serine protease targets in glomerulonephritis. We investigated whether PAR1/2 are required for alterations in GEC phenotype that are mediated by PR3 or elastase during active glomerulonephritis. Endothelial PARs were assessed by flow cytometry. Thrombin, trypsin and agonist peptides for PAR1 and PAR2, TFLLR-NH_2_ and SLIGKV-NH_2,_ respectively, were used to assess alterations in PAR activation induced by PR3 or elastase. Endothelial von Willebrand Factor (vWF)release and calcium signaling were used as PAR activation markers. Both PR3 and elastase induced endothelial vWF release, with elastase inducing the highest response. PAR1 peptide induced GEC vWF release to the same extent as PR3. However, knockdown of PARs by small interfering RNA showed that neither PAR1 nor PAR2 activation caused PR3 or elastase-mediated vWF release. Both proteases interacted with and disarmed surface GEC PAR1, but there was no detectable interaction with cellular PAR2. Neither protease induced a calcium response in GEC. Therefore, PAR signaling and serine protease-induced alterations in endothelial function modulate glomerular inflammation via parallel but independent pathways.

## Introduction

Human neutrophils engulf, digest and promote extracellular killing of invading microorganisms. This function is aided by the release of the serine proteases, PR3 and elastase, and by the formation of serine protease-containing neutrophil extracellular traps (NETS) [1]. Clinical and experimental findings also indicate a key role for these released serine proteases during inflammation. Elevated plasma levels of PR3 and elastase are detected during the active inflammatory phase of several chronic diseases [2], [3]. Within the kidney, PR3 and elastase containing NETs have been detected in human glomeruli, affected by inflammatory processes [4] with inefficient NET dismantling implicated in renal damage [5]. At the cellular level, the release of serine proteases potentially induces injury and/or modulates cell responses via cleavage of soluble, cell-surface [6] or intracellular proteins [7]. Indeed, infusion of neutrophil serine proteases, such as elastase, through renal arteries leads to localization of the enzyme on the glomerular capillaries and transient proteinuria [8]. Both PR3 and elastase have been specifically implicated in the glomerular endothelial cell (GEC) activation/injury that occurs during vasculitic glomerulonephritis. In this disorder, autoantibodies develop that target neutrophil PR3 or myeloperoxidase. Binding of target autoantigens at the neutrophil surface leads to enhanced neutrophil-endothelial adhesion [9] and protease release [4]. *In-vitro* treatment of endothelial cells with serine proteases (1–5 µg/ml) has been shown to induce a behavioral shift towards to a more pro-adhesive and proinflammatory phenotype within endothelial cells and HUVEC [10]. Taken together, these findings suggest a direct link between serine protease release and renal disease, regulated at the endothelial level.

The purpose of this study was to evaluate the role of protease activated receptors (PARs) in serine protease mediated responses, including release of endothelial von Willebrand factor (vWF), in the context of glomerular inflammation. PARs are seven-trans-membrane G-protein coupled signaling proteins that are activated by proteolytic cleavage, producing a tethered binding ligand [11]. The original search for PAR1 and PAR2 receptors was driven by investigating the cellular actions of thrombin [11], [12] and the PAR1-independent action of trypsin respectively [13]. Thrombin and trypsin, via PAR activation, have a variety of cellular effects [14], [15], including endothelial stimulation with up-regulated tissue factor expression and Weibel Palade body mobilization resulting in surface P-selectin expression and vWF release [16]–[18]. PAR signaling induces this Weibel Palade body exocytosis via a calcium and cdc42-dependent mechanism [19]. PAR1 protein is expressed by renal tissue [20] while elevated PAR2 has been detected in inflamed renal tissue [21]. PAR2 activation can also induce human proximal tubular cell [22] and mesangial cell proliferation [23], with the latter implicated in the development of mesangioproliferative glomerulonephritis [24]. *In-vivo* models of crescentic glomerulonephritis indicate that both PAR1 (−/−) and PAR2 (−/−) deficient mice have reduced crescent formation and serum creatinine concentrations [25], [26].

PAR1 signaling in the context of pro-inflammatory role of thrombin-mediated effects has been extensively studied. However, recent studies have demonstrated important roles in resolution such that anti-inflammatory, antithrombotic and renoprotective activity results from an association of activated protein C (APC), its endothelial-bound receptor (EPCR) and surface PAR1 [27]–[29]). PR3 has been shown to inactivate, endothelial-bound EPCR [30], but the exact nature of any direct interaction of PR3 or elastase with surface PAR1 on glomerular endothelial cells has not been clearly defined. Thus, this study investigated the influence of PR3 and elastase on proteolytic cleavage of glomerular endothelial PAR-mediated vWF release.

## Materials and Methods

### Ethics Statement

The protocol used for this study was approved by South Birmingham Research Ethics Committee and Walsall Local Research Ethics Committee.

### Materials

Specific PAR agonist peptides for (i) PAR1 (TFLLR-NH_2_) or (ii) PAR2 (SLIGKV-NH_2_) were supplied by Peptide International. The serine proteases used were PR3, elastase, thrombin and trypsin. PR3 was used at concentrations between 1–5 µg/ml (RMM = 29 kd, 34.5–172.4 nM), Athens Research and Technology Cat. no. 16-14-161820. The specific activity of PR3 was 16 µmol of p-nitrophenol/mg of PR3/min at room temperature using t-butyloxy carbonyl p-nitrophenylester (Boc-Ala-OPhNO_2_) as a substrate. Human neutrophil elastase was also used at concentrations between 1–5 µg/ml (RMM = 29.5 kd, 33.9–169.5 nM), Calbiochem Cat. no. 324681. The specific activity was 20 units/mg of elastase, where one unit is defined as the amount of enzyme that will hydrolyze 1 µmol of MeO-Suc-Ala-Ala-Pro-Val-*p*NA (Cat. no. 454454) per min at 25°C, pH 8. The same concentration range of PR3 and elastase stated above was used in a parallel study [10]. Thrombin from human plasma was used at ≈10 or 100 nM (RMM = 37.4 kd, 10 units/ml, therefore 1 unit/ml ≈10 nM), Sigma Cat. no. T6884. Thrombin concentration was determined using platelet P-Selectin expression and EC vWF release. The specific activity for thrombin was 2,000 NIH units/mg of protein. Trypsin was used at 50 nM (RMM = 23.8 kd, ≈23.8 units/ml, therefore 1 unit/ml ≈2.1 nM), Sigma Cat. no. T0303. 50 nM produced a rapid, detectable, reproducible and sub-maximal response and was therefore chosen for subsequent experiments. The specific activity of trypsin-1G Type IX-S was between 13,000–20,000 BAEE units/mg of protein. One BAEE unit will produce a ΔA_253_ of 0.001 per min at pH 7.6 at 25°C using BAEE as substrate.

Antibodies used were PE-labeled SPAN12 (Cat. no. IM2583) and WEDE15 (Cat. no. IM2584) monoclonal antibodies (Immunotech, Beckman Coulter ‘CoulterFlow’), anti-PAR2 antibody, SAM11 (Santa Cruz) and rabbit anti-human vWF polyclonal antibodies (DAKO). Stealth™ RNAi used were PAR1 (F2R code: HSS103468) and/or PAR2 (F2RL1 code: HSS103471) or negative control non-silencing Stealth™ RNAi (siRNA control). All siRNA reagents were supplied by Invitrogen.

### Cells

Umbilical cords were obtained with informed consent from Birmingham Women’s Hospital. Human umbilical vein endothelial cells (HUVEC) were then isolated and cultured as described [31]. Conditionally immortalized human glomerular endothelial cells (GEC) were maintained in supplemented endothelial basal medium-2 (Lonza) (a gift from Dr S. Satchell, Bristol, UK) [32]. Human embryonic kidney cells, HEK-293 were an established cell line [33]. These cells were maintained in 10% FBS (Sigma), 2 mM glutamine and 100 U/ml penicillin and 100 µg/ml streptomycin (Invitrogen). HEK-293 were used because they constitutively express both PAR2 and PAR1 and produce a PAR agonist-mediated calcium signal, then rapidly (<10 min) replenish their intracellular calcium stores [34].

### Real Time RT-PCR

RNA was isolated from cells using a Qiagen RNeasy Mini Kit 50 (Qiagen) and real-time RT-PCR was performed using a QuantiTect probe RT-PCR kit according to the manufacturer’s recommendations (Qiagen). Briefly, RNA (10 ng) was added to: QuantiTectTM probe and reverse transcriptase master-mixes; β-actin VIC-labeled primers/probes (Applied Biosystems); with either PAR1 (Assay ID: Hs00169258_ml) or PAR2 FAM labeled primers/probes (Assay ID: Hs00608346_ml Applied Biosystems). Samples were amplified for 35 cycles and analyzed using a 7500 Real-Time PCR machine (Applied Biosystems). The relative expression units (REU) were determined using β-actin as a control. Changes in mRNA expression in treated cells relative to their controls (Relative quantity, RQ) were also determined.

### PAR1 or PAR2 Knockdown by siRNA Treatment

Confluent EC were incubated for 4 hr in: (i) Optimem medium alone or Optimem medium containing (ii) 0.2% Lipofectamine RNAiMax (LF control) plus 20 nM of Stealth™ RNAi for silencing (iii) PAR1 and/or (iv) PAR2 or (v) non-silencing Stealth™ RNAi. After siRNA treatment, the medium was replenished with an equal volume of supplemented Medium 199 without antibiotics. Cells were then incubated in this medium for 48–72 hr before use in subsequent assays.

### Endothelial Cell vWF Expression

Isolated or cultured EC were shown to express von Willebrand Factor (vWF) [35]. VWF release was assessed by sandwich ELISA using anti-vWF antibodies for both capture (unconjugated) and detection (HP-conjugated).

### Calcium Measurement by Fluorescent Microscopy

GEC were seeded into gelatin-coated 8-well borosilicate chambered coverglass wells (Thermo Fisher Scientific) at 4×10^4^ GEC/well. Confluent cells were labeled with 40 µM fura-2-AM ester for 40 min and then washed with HBSS with 1% HEPES buffer (HBH). Pairs of fluorescence images at two excitation wavelengths (high calcium-380 nm) and (no calcium-340 nm) produced a fluorescence ratio image as a direct measure of cytoplasmic calcium changes. Pairs of images were recorded at 3 s intervals for 60 s before the addition of a stimulus to produce a baseline value, then recorded every 1–3 s for a further 6 min. Mean fluorescence ratios for 30 adherent cells per treatment were calculated from the 340/380 nm ratio after outlining of each cell. Peak rise data for each cell were calculated by subtracting a baseline value for each ratio. Mean peak rise values for all 30 cells were calculated to produce peak rise per condition. The calcium concentration (nM) was then determined from the peak rise values. Fluorescent images were captured and analyzed using a fluorescent inverted microscope (Leica) and SimplePCI software (Hamamatsu Corp.).

### Calcium Measurement by Spectrofluorimetry

Adherent EC were incubated for 2 hr in Medium 199+0.15% BSA alone or 5 µg/ml of PR3 or elastase. Cells were loaded with 1 µM Indo-1 AM ester (Invitrogen) for 40 min and then harvested and resuspended at 1×10^6^ cells/ml in Ca^2+^ Hanks’ balanced salt solution (HBSS Sigma) +25 mM HEPES buffer (Sigma). The ratio of the fluorescent intensity at the two emission wavelengths (495 nm (Ca^2+^ free) and 405 nm (Ca^2+^) was observed for 120 s producing a stable baseline, and then a stimulus was added through a stopper in the top of the fluorimeter. After addition of the stimulus, the trace was observed until a stable plateau response was achieved. The ratio of fluorescence intensity for maximum and minimum calcium response was determined using ionomycin (5.6 µM) and EGTA (3 mM), respectively, allowing the generation of individual calibration files for each experiment to calculate Ca^2+^ mobilization as previously described [36]. The change in intracellular calcium levels induced by a particular stimulus was determined by subtracting the baseline value (mean calcium value over first 110 s) from all calcium values. Data were recorded using a luminescence spectrofluorimeter and FL Winlab software (Perkin Elmer). Note: Calcium data were expressed as either calcium concentration in nM in cells in suspension or as fluorescence ratio (340/380 nm) peak rise data or as a percentage of the fluorescence ratio produced by a positive control.

### Flow Cytometry

Cells were harvested with cell dissociation buffer (Sigma) and resuspended at 1×10^6^ cells in PBS with 5% FBS. Surface PAR1 protein expression was assessed using PE-labeled SPAN12 and WEDE15 mouse monoclonal antibodies with an appropriate PE-labeled isotype control. The SPAN12 monoclonal antibody recognized amino acid residues _35_NATLDPR_41_/_42_SFLLR_46_, spanning the PAR1 thrombin cleavage site [37]. SPAN12 therefore detected only uncleaved PAR1 receptors. WEDE15 monoclonal antibody recognized the site _51_KYEPFWEDEEKNES_64_ where thrombin binds to PAR1 [38]. Reduced WEDE binding indicates removal of either (i) the thrombin binding site or (ii) the entire receptor from the cell surface.

PAR2 protein expression was determined by using a Fixation/Permeabilization buffer (eBioscience), followed by (i) SAM11, a mouse anti-PAR2 monoclonal antibody, raised against _37_SLIGKVDGTSHVTG_50_ or (ii) an isotype control antibody, diluted in a permeabilization buffer (eBioscience). 10,000 events were acquired using a BD FACS Calibur. Data were analyzed using Cell Quest software (BD biosciences).

### Detection of PAR1 Internalization Using Flow Cytometry

Adherent HUVEC were pre-incubated in Medium 199 with 0.15% BSA for 30 min with 0.02%v/v DMSO (vehicle) or with an internalization inhibitor: Dynasore (50 µM, Sigma). http://www.ncbi.nlm.nih.gov/pubmed/16740485Dynasore inhibits endocytosis by acting on a small GTPase called dynamin, which normally releases endocytic vesicles from the cell membrane [39]. Internalization was also inhibited by maintaining cells at 4°C. HUVEC were then incubated in the presence of Dynasore (37°C) or on ice with 5 µg/ml of either PR3 (172 nM) or elastase (169.5 nM) for 2 hr. Thrombin (10 U/ml) induced PAR1 internalization was used as a positive control. The extent to which PR3 and elastase removed surface PAR1 by internalization was detected by WEDE antibody binding using flow cytometry (see above), under these inhibitory conditions.

### Statistical Analysis

Graphs were produced and statistical analysis performed using GraphPad Prism software. Paired T-tests were used to compare two matched variables. Differences among groups were analyzed using one-way analysis of variance, followed by Dunnett post tests and, where appropriate, two-way analysis of variance was also used. A probability of 0.05 or less was considered significant. Data were expressed as means ± standard error of the mean for at least three independent experiments.

## Results

### Endothelial vWF Release Induced by PR3, Elastase or Specific PAR-ap’s

**Figure 1:**
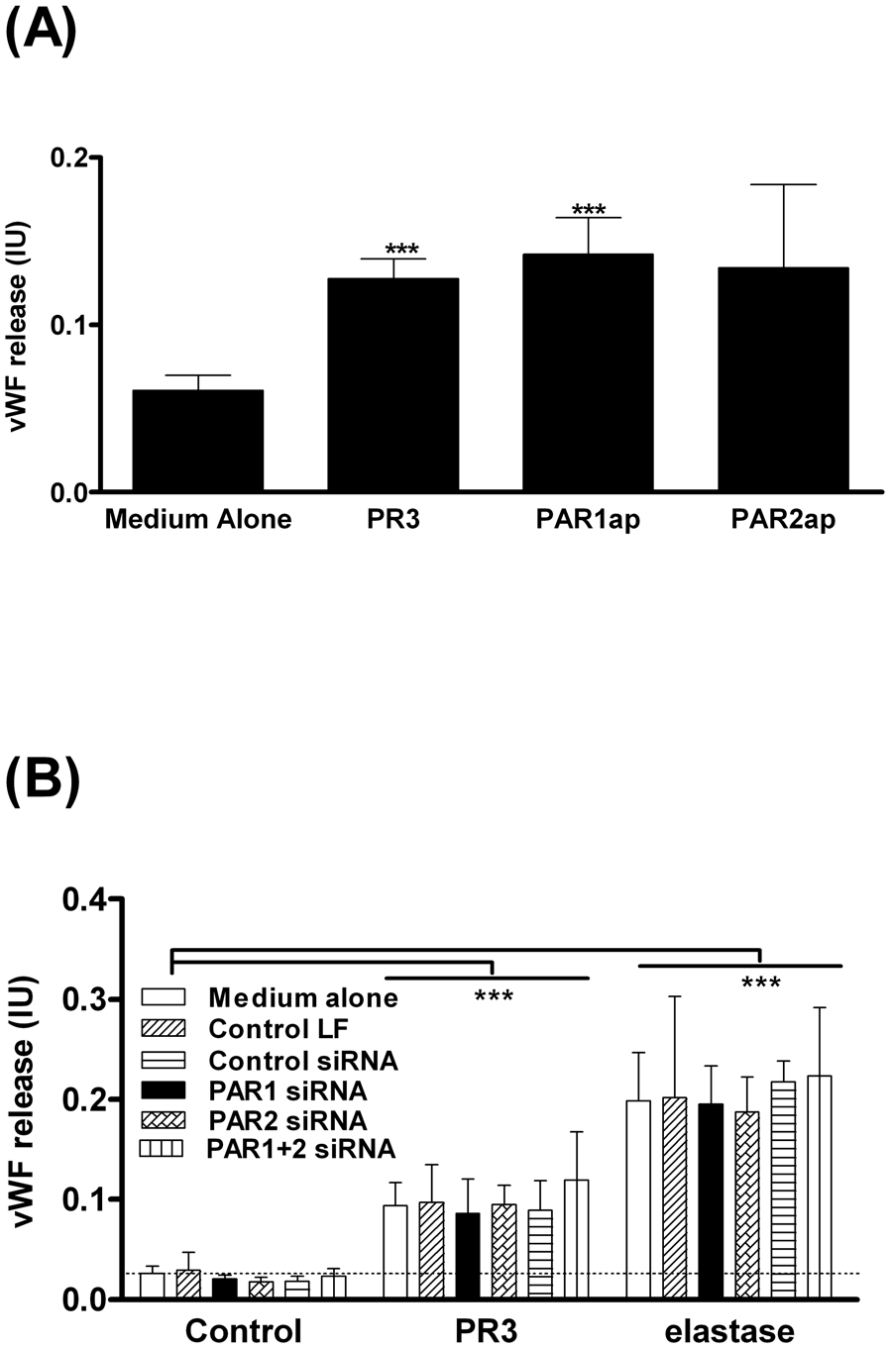
PR3 and elastase induce vWF release via a PAR-independent mechanism. vWF release from untreated GEC in medium alone and endothelial cells after exposure for 2 hr to 100 µM PAR1ap (TFLLR-NH_2_) or 100 µM PAR2ap (SLIGKV-NH_2_) or 1 µg/ml (34.5 nM) PR3 was measured (Fig. 1A). Data were expressed as mean ± SEM, n = 4. Statistic tests used for Fig. 1A were paired T-tests. For the siRNA knockdown experiments of Fig. 1B, confluent GEC were incubated for 4 hr. Three controls were used (1) Optimem medium (Medium alone; open bars) or (2) Optimem medium containing 0.2% Lipofectamine RNAiMax without siRNA (Control LF; diagonal line bars) or with (3) 20 nM non-silencing scrambled Stealth™ RNAi (Control siRNA; horizontal line bars). PAR expression was silenced by using 20 nM of Stealth™ RNAi against PAR1 (PAR1 siRNA; black bars), or PAR2 (PAR2 siRNA; cross-hatched bars), or both PAR1 and PAR2 (PAR1+2 siRNA; vertical line bars). The vWF release induced by 1 µg/ml PR3 (34.5 nM) or elastase (33.9 nM) were assessed 48 hr after siRNA treatment (Fig. 1B). Statistic test used for Fig. 1B was a Two-way ANOVA p = 0.0001 comparing vWF release from untreated cells vs. cells treated with PR3 and elastase under all four siRNA conditions.

Using endothelial vWF release from viable cells as a read-out, we determined whether the cleavage of PAR by serine proteases directly activated GEC. Both PR3 (1 µg/ml) and the PAR1 agonist peptide (PAR1ap; TFLLR-NH_2_; 100 µM), induced vWF release that was more than double the release observed with controls, (n = 4; paired t–tests p = 0.0073 for PR3 and p = 0.0338 for PAR1ap (Fig. 1A)). PAR2 agonist peptide (PAR2ap; SLIGKV-NH_2_; 100 µM) induced vWF but this was not statistically significant.

Despite the equivalent GEC responses to PAR1ap and PR3 as shown in Fig. 1A, siRNA knock-down of PAR1, PAR2 or both PAR1 and PAR2 did not alter either PR3-or elastase-induced vWF release (Fig. 1B). There was no detectable effect of siRNA treatment on endothelial monolayer integrity (Fig. S1A). Successful PAR1 and PAR2 knockdown was confirmed at the transcriptional levels by RT-PCR (Fig. S1B) and translational levels by flow cytometry (Fig. S1C for PAR1 and Fig. S1D for PAR2). PAR knockdown was also confirmed by loss of PAR agonist peptide activity (Fig. S1E). PAR-ap-induced vWF release was also reduced after PAR knockdown (data not shown). Elastase-and PR3-induced vWF release was abolished in the presence of the serine protease inhibitor, alpha-1 anti-trypsin (Fig. S2) indicating that serine protease induced vWF is solely dependent on proteolytic activity. These data indicate that both PAR agonists and leukocyte proteases induced vWF release, but via independent mechanisms.

### PAR1 Cleavage from the Endothelial Surface by PR3 or Elastase

**Figure 2:**
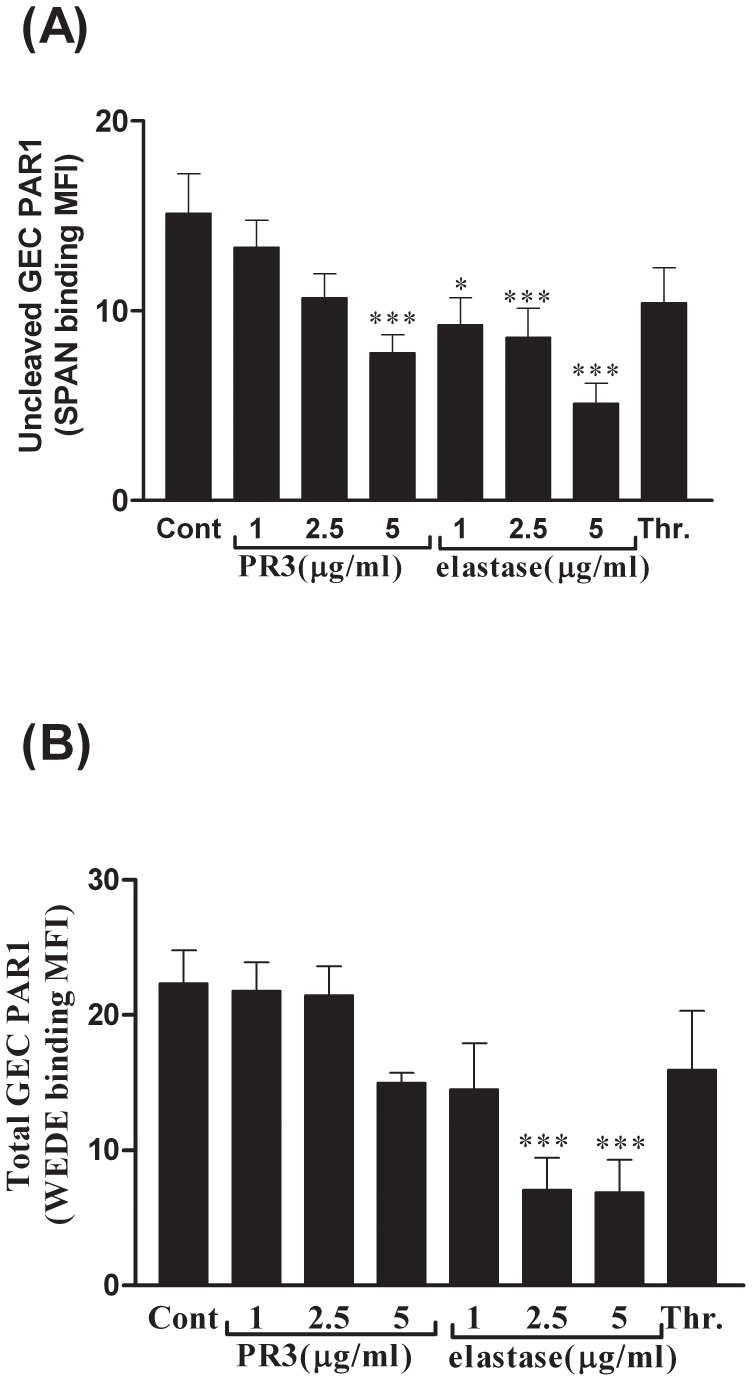
PAR1 cleavage from the endothelial surface by PR3 or elastase. Cleavage of GEC surface PAR1 was detected using two specific antibodies SPAN (Fig. 2A) and WEDE (Fig. 2B) on cells harvested after 2 hr exposure to thrombin (1 U/ml, ≈10 nM) or 1–5 µg/ml (34.5–172 nM) of PR3 or 1–5 µg/ml elastase (33.9–169.5 nM). Data were expressed as mean ± SEM, n = 3. One-way ANOVAs with Dunnett’s post tests were employed to assess statistical significance.

After detecting PR3-or elastase-mediated endothelial vWF release which was independent of PAR1 (and PAR2) signaling, we investigated whether these proteases were (as predicted) directly interacting with glomerular endothelial PAR1. Cleavage of PAR1 by PR3 and elastase on the surface of GEC was assessed by flow cytometry. Elastase (tested at 1–5 µg/ml) directly interacted with and cleaved GEC PAR1 resulting in a loss of SPAN antibody binding (the thrombin cleavage site), and also a loss of downstream WEDE antibody binding (Fig. 2A and 2B). This reached significance at 2.5 µg/ml (n = 3, one-way ANOVA with Dunnett’s post test p<0.05 for cleavage and p<0.001 for total surface expression*)*. GEC were also sensitive to cleavage by PR3 (tested at 1–5 µg/ml) causing a predominantly ‘thrombin-like’ PAR1 cleavage with loss of SPAN binding that was significant at 5 µg/ml *(*n = 3, one-way ANOVA, p = 0.0012 with Dunnett’s post test p<0.001 for cleavage 5 µg/ml vs. control; Figs. 2A and 2B). Using this flow cytometric methodology, thrombin (10 U/ml, ∼100 nM) produced a similar pattern of SPAN and WEDE antibody binding as PR3 (5 µg/ml, 172 nM; Fig. 2A and 2B).

**Figure 3:**
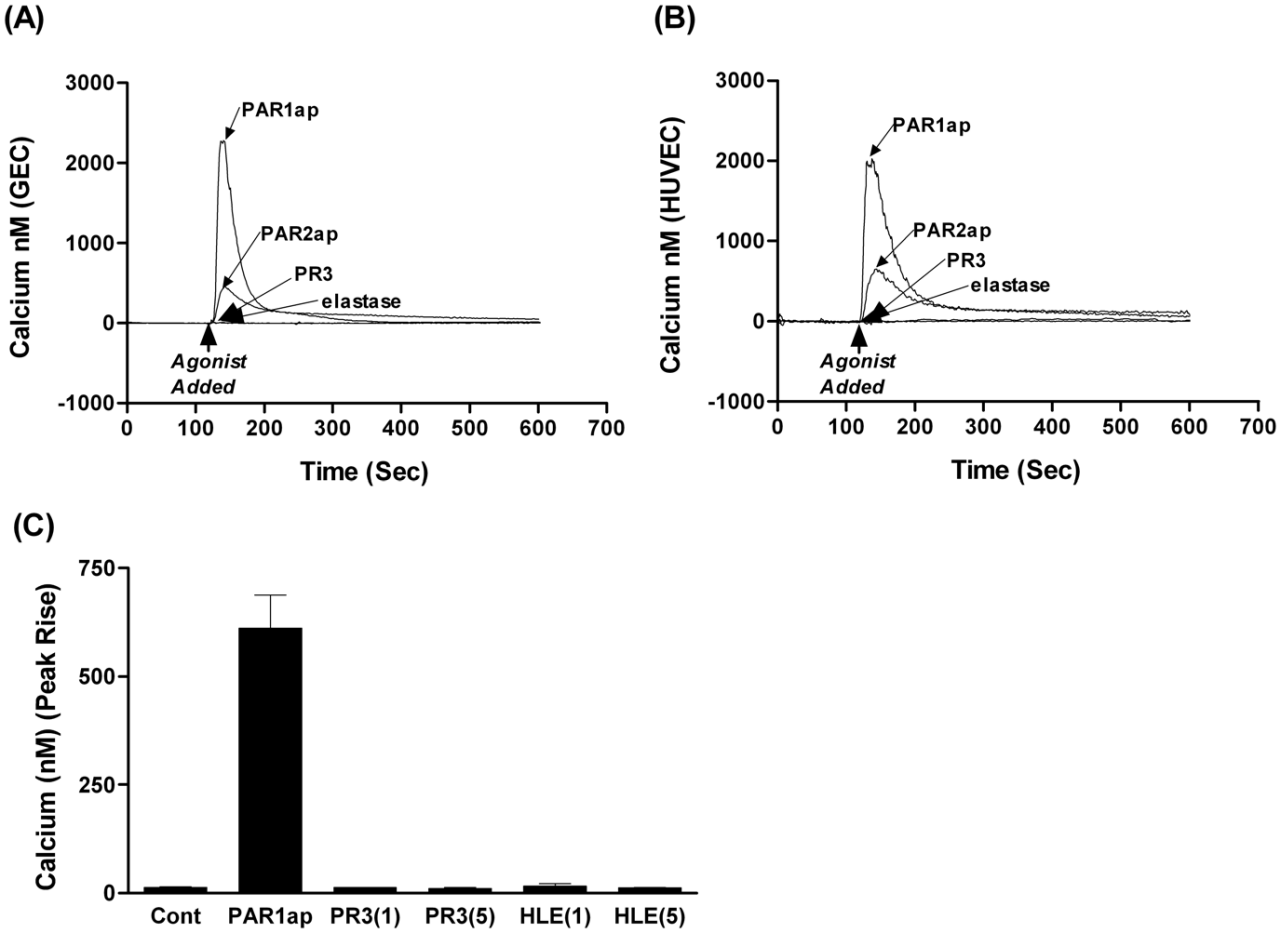
Effect of PR3 or elastase on EC Ca^2+^ signaling. Fig. 3 shows the inability of 5 µg/ml of PR3 (172 nM) or elastase (169.5 nM) to induce a calcium signal in endothelial cells in suspension whether GEC (Fig. 3A), HUVEC (Fig. 3B) or adherent GEC (Fig. 3C). The presence of functional PAR receptors on the surface of these cells was confirmed using PAR1ap and PAR2ap were used as positive controls (Fig. 3A–C). Fig. 3A& B show representative traces from a single experiment. Fig. 3C shows data from 7–10 independent experiments (mean ± SEM).

To determine whether any loss in WEDE antibody binding to surface PAR1 was due to receptor internalization or extensive cleavage, PAR1 internalization was inhibited (i) under cold conditions at 4°C or (ii) with a 30 min pre-incubation with Dynasore (50 µM, an inhibitor of dynamin-regulated endocytosis [40], [41]). Inhibiting internalisation with temperature (4°C) abolished the PR3-induced loss in WEDE antibody binding to surface PAR1. This indicates that PR3 (5 µg/ml, 172 nM) treatment induced cleavage of surface PAR1 and internalisation of the receptor (Fig. 3A). In contrast, elastase (5 µg/ml, 169.5 nM) was able to reduce WEDE binding even under conditions where the internalization process was impaired. This suggests that elastase treatment also induced internalisation of the PAR1 receptor but a component of elastase-mediated removal of WEDE binding was due to extensive cleavage (Fig. S3A). Dynasore, an inhibitor of a later stage of the internalisation, partially inhibited protease-induced loss in WEDE binding to surface PAR1 (Fig. S3B).

### Effect of PAR Peptides or Leukocyte Proteases on EC Calcium Signaling

To determine whether PR3 or elastase induced downstream signaling processes, we measured changes in cytoplasmic calcium in response to the proteases in unstimulated ECs. In addition we assessed the ability of these proteases to alter subsequent PAR1 or PAR2 receptor activation with cells in suspension and adhered to a surface using spectrofluorimetry and fluorescent microscopy. Neither PR3 nor elastase induced downstream calcium signaling in either GEC (Fig. 3A) or HUVEC (Fig. 3B) in suspension or in adherent GEC (Fig. 3C). The inability of PR3 or elastase to induce a calcium signal was not restricted to EC, as they were also unable to provoke a calcium response in human kidney cells, HEK-293 (data not shown).

Both endothelial types, HUVEC and GEC, produced comparable calcium signals, when stimulated by PAR1ap and PAR2ap (Fig. 3A and 3B). The magnitude of EC response induced by specific PAR1ap was greater than that induced by PAR2ap (Fig. 3A and 3B). Using EGTA to remove extracellular calcium indicated that these PAR1/2ap-induced signals were partly due to mobilization of intracellular calcium stores (data not shown).

**Figure 4:**
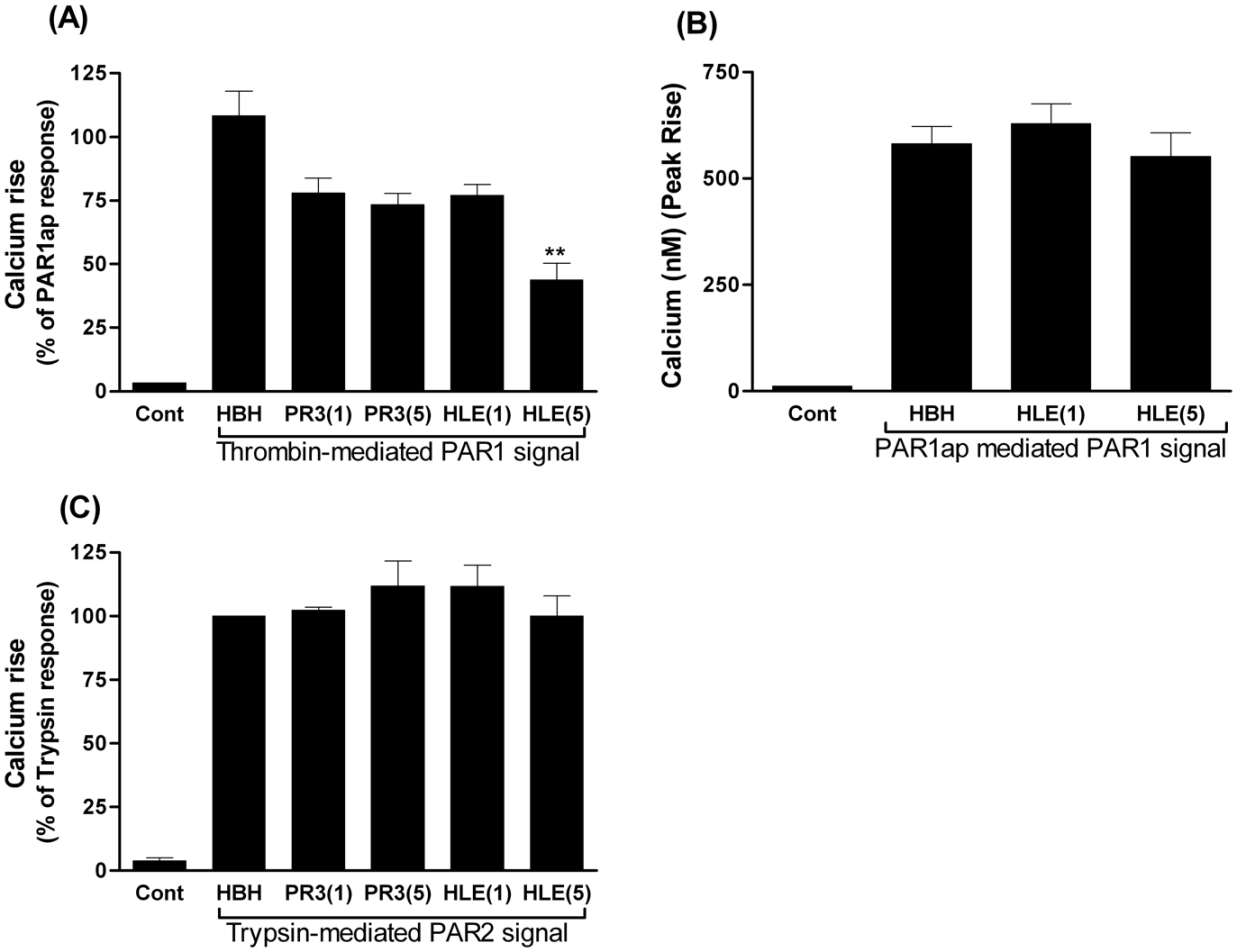
The immediate effect of PR3 or elastase on PAR signaling induced by other stimuli. Fig. 4A–C show the effect of a 10 min pre-treatment of GEC with PR3 (34.5 nM = 1 µg/ml PR3(1) or 172 nM = 5 µg/ml PR3(5)) or elastase (33.9 nM = 1 µg/ml HNE(1), or 169.5 nM = 5 µg/ml HNE(5)), on subsequent PAR1 calcium signaling induced by either thrombin (Fig. 4A) or PAR1ap (Fig. 4B) and on subsequent trypsin activation of PAR2 (Fig. 4C). Baseline calcium levels in untreated cells were recorded (control). The bars marked ‘HBH’ show responses after addition of buffer alone (Hanks balanced salt solution +20 mM HEPES) in the absence of any protease during a 10 min pre-treatment period, followed by stimulation of the cells with either thrombin (Fig. 4A) or PAR1ap (Fig. 4B) or PAR2 (Fig. 4C) were used as positive controls for each experiment. Fig. 4A–C show data from 3–4 independent experiments (mean ± SEM).

Pre-treatment of GEC with either PR3 or elastase (1–5 µg/ml for 10 min) induced a concentration-dependent inhibition of thrombin-mediated PAR1 receptor activation (Fig. 4A). Elastase induced a greater inhibition of subsequent thrombin activation than PR3 at 5 µg/ml, after 10 min (Fig. 4A). However, elastase, at this early time point, did not alter PAR1ap-mediated PAR1 receptor activation (Fig. 4B), indicating that elastase induced early removal of the thrombin cleavage site, without affecting the agonist peptide binding site. Neither PR3 nor elastase caused immediate inhibition of trypsin-mediated PAR2 receptor signaling indicating that trypsin cleavage site of PAR2 was unaffected by these proteases (Fig. 4C).

**Figure 5:**
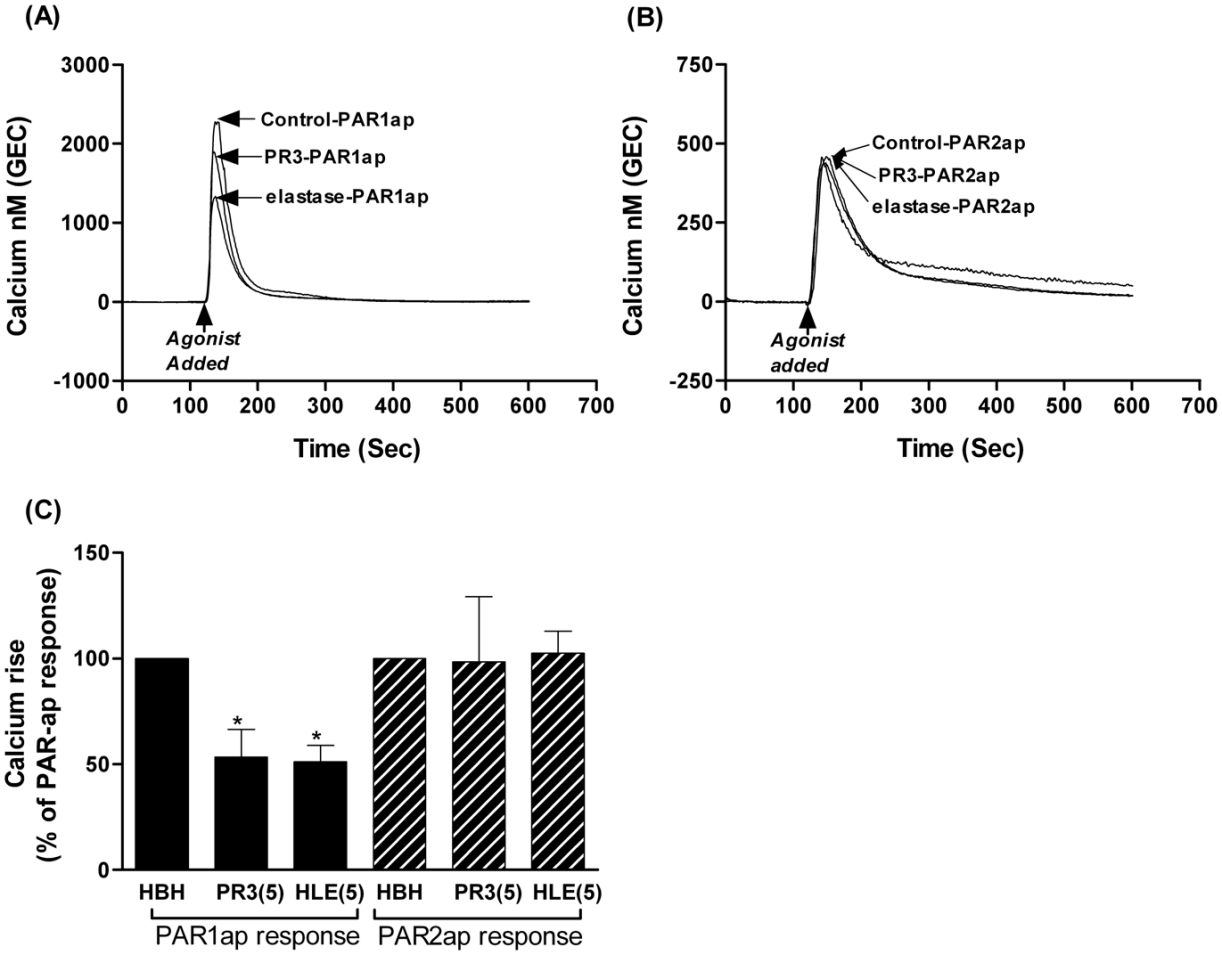
The chronic effect of PR3 or elastase on PAR signaling induced by other stimuli. Fig. 5A–C show the effect of a 2 hr pre-treatment of GEC with 5 µg/ml of PR3 (172 nM) or elastase (169.5 nM, HNE), on subsequent PAR1ap mediated PAR1 signaling (Fig. 5A,C) and PAR2ap mediated PAR2 signaling (Fig. 5B,C). Fig. 5A& B show representative traces for a single experiment. Fig. 5C shows data from 4 independent experiments (mean ± SEM).

Persistent exposure to either PR3 or elastase (5 µg/ml for 2 hr) resulted in partial inhibition of PAR1ap-induced GEC PAR1 receptor signaling (Fig. 5A and 5C), indicating that both proteases (over longer periods) were able not only to remove the thrombin cleavage site but also to reduce agonist peptide binding, consistent with the flow cytometry observations using antibody detection of SPAN and WEDE binding sites. Conversely, neither PR3 nor elastase, over a 2 hr period, inhibited GEC PAR2ap-induced PAR2 receptor activation (Fig. 5B and 5C).

## Discussion

### PR3 and Elastase Disarm Glomerular Endothelial PAR1 Receptor

In the kidney, activation of PAR1 is known to either induce cellular injury via pro-inflammatory signaling or cytoprotection by promoting an anti-inflammatory pathway [26]–[28]. Here we have demonstrated PR3-and elastase-induced vWF release which was independent of PAR activation. This was shown by siRNA knockdown experiments. As observed in other cell types [34], these proteases also negatively regulated glomerular endothelial PAR1 signaling. This was demonstrated by their failure to elicit calcium signals in GEC and also by their ability to block PAR1 activation by its agonists, thrombin and activating peptide. The cleavage and inactivation of PAR1 by elastase and PR3 were both time-and concentration-dependent. The inability of PR3 or elastase to induce vWF release via PAR cleavage may be due to the absence of a calcium signaling. This is supported the Klarenbach 2003 study which demonstrated that PAR1ap-and PAR2ap-induced vWF release is calcium dependant [19].

The region of the PAR1 extracellular domain that was close to the thrombin cleavage site was most susceptible to cleavage by elastase and PR3; this was demonstrated by initial inhibition of the thrombin response, followed by inhibition of the peptide agonist response. However, we do not anticipate cleavage of the thrombin activation site (R^40^S^41^) by these proteases as this cleavage would release a new tethered ligand, starting with SFLLR, that would result in receptor activation [42]. Identification of the precise cleavage site(s) would require a site-directed mutagenesis approach, similar to the one used for PAR2, to elucidate this [43].

Both PR3 and PAR1ap triggered equivalent levels of vWF release, which compliments previous observations by Steppich et al (2008) in which PAR1ap appeared to mimic the effect of PR3 in triggering tissue factor mRNA expression [4]; comparable levels of PR3 and elastase mediated cleavage of endothelial PAR1 receptors were also obtained [4]. Serine proteases bind to their target protein and cause either cleavage at a specific site resulting in activation or, alternatively, inappropriate or multiple site cleavage resulting in disarming of the receptor [44]. Steppich et al (2008) and our current findings could, therefore, be interpreted in three ways: (i) PR3, but not elastase, resulted in activatory PAR1 receptor cleavage; (ii) PR3, but not elastase-mediated PAR1 cleavage produced a free activating peptide, capable of activating other PAR1 receptors or (iii) both PR3 and elastase caused inhibition of subsequent receptor signaling, in the absence of an initial signal, (i.e. receptor disarming (Fig. S4)).

Using calcium signaling, we have clearly demonstrated that the cleavage of glomerular endothelial PAR1 receptors by both PR3 and elastase resulted in PAR1 receptor disarming that was both time-and concentration-dependent. Short term exposure of GEC to either protease (1–5 µg/ml, (≤172 nM), 10 min) caused the removal of the PAR1 activating peptide sequence for a minority of cells without affecting the binding site of that activating peptide. Long term exposure to elastase and PR3 (5 µg/ml, (≤172 nM), 2 hr), resulted in reduced PAR1ap-mediated signaling, indicating that after more prolonged exposure the proteases affected not only the thrombin cleavage site of the PAR1 receptor but also affected the binding of the free activating peptide. It is important to put these observations in context and not over emphasis our findings. Recent studies have suggested that the regulation of PARs is not straightforward and is both agonist-dependent and cell type–specific [45]. PARs have the ability to regulate opposite effects dependent on their agonist, location (i.e. within caveolae) and associated binding partners (Biased signaling) [46]. Others have shown serine protease-induced apoptosis potentially triggered by PAR1 signaling [47]. These findings may therefore imply the inhibition of one major calcium dependent PAR1 signaling pathway but not the destruction of all potential PAR1 signaling routes.

In a wider context, PR3 and elastase appear not only to directly affect PAR1 activity, but also increase coagulation in-vivo [48] by removing cell-surface tissue factor pathway inhibitor (TFPI) [49]. PR3 can also cleave endothelial surface-bound EPCR, reducing APC generation. This targeting of both PAR1 and its anti-inflammatory regulators, EPCR and APC, would abrogate the PAR 1-dependent barrier-protective response in endothelial cells [50], potentially resulting in a shift away from resolution and towards persistent inflammation.

In this study, the neutrophil serine proteases PR3 and elastase did not regulate PAR2 calcium mobilization within GEC that express functional PAR2, as indicated by the failure of these proteases to either activate or disarm PAR2. Elegantly designed studies by Ramachandran and colleagues indicated an alternative route of PAR2 activation, circumventing calcium signaling but activating MAP kinase signaling [51], [52]. They observed both PAR2 disarming and the capacity of elastase (but not PR3) to activate this alternative pathway [51]. PAR surface expression, cleavage and regulation of signaling (e.g. protease-induced disarming) can also be affected by the factors such as N-linked glycosylation [53], [54]. Those studies are comparable with our present study, because the same source of trypsin was used; however, the sources of both PR3 and elastase were different. Unlike PAR1, we were unable to detect any glomerular endothelial PAR2 disarming. Initially, we considered that these observed differences in PAR2 cleavage were due to the use of synthetic or recombinant polypeptides instead of an intact cell expression system to study this phenomenon. Further, Al-Ani and Hollenberg (2003) observed that cellular PAR2 was resistant to extensive downstream cleavage by serine proteases [43]. However, cell surface PAR2 disarming has been detected elsewhere. PAR2 inhibition by elastase has been previously reported in epithelial cells [55], while retracted reports support a role for PR3 in PAR2 receptor signaling in both epithelial [56] and non-epithelial cells [57]. Epithelial cells express functional PAR1, PAR2 and PAR-4, with PAR2 acting as the dominant PAR, inducing the strongest cellular response with respect to cytokine production [58]. In contrast, we observed that GECs have a higher cellular response to PAR1 activation than PAR2. Findings of another study in human and mouse leukocytes also discount a role for PR3 in PAR2 activity [59]. The apparent differences in the outcome of these studies may be due to the cell types employed.

In conclusion, neutrophil-derived serine proteases PR3 and elastase bind to, and directly modulate, multiple protein targets on the surface of GEC including PAR1, with no inhibitory effect on PAR2, the member of the PAR family upregulated during inflammation. Calcium signaling-independent modulation of endothelial function leads to the pro-inflammatory, pro-thrombotic release of proteins such as vWF, while the disarming of the glomerular endothelial PAR1 receptor may abrogate any anti-inflammatory, protective effects that could be supported by this receptor. Modulation of serine protease activity, rather than direct modulation of PAR receptors, could be tissue protective during the acute phase of some glomerulonephritides, such as vasculitic diseases where neutrophil activation and protease release is prominent.

## Supporting Information

Figure S1 **PAR1 and PAR2 mRNA and protein knockdown by siRNA.** Fig. S1A shows phase contrast photomicrographs of (i) untreated HUVEC and cells exposed to (ii) non-targeting siRNA (Control siRNA) and (iii) PAR1 siRNA and (iv) PAR2 siRNA. PAR1 (black) and PAR2 (grey) of GEC mRNA levels were abolished by siRNA treatment. This silencing of PAR mRNA was detected, 48 hr after a 4 hr siRNA treatment. Data were expressed as relative expression compared to lipofectamine treated cells (Fig. S1B n = 5–6). The reduction in PAR1 (Fig. S1C n = 3) and PAR2 (Fig. S1D n = 3) protein levels induced by siRNA treatment was detected by flow cytometry using WEDE (anti-PAR1) and SAM11 (anti-PAR2) antibodies. The reduction in glomerular endothelial cell PAR1 (Fig. S1E) and PAR2 (Fig. S1F) calcium signal due to siRNA knockdown was also assessed. These are representative calcium traces (n = 3, for PAR1 p = 0.0493*, for PAR2 p = 0.0081**).
(TIF)

Figure S2 **PR3 or elastase induced GEC vWF release in the presence of alpha anti-trypsin.** Fig. S2 shows that GEC vWF release in response to PR3 or elastase. This is abolished in the presence of alpha anti-trypsin (+α1AT). The statistical symbols indicated significant difference either compared to *untreated controls (Cont.) or ^+^compared to protease treatment only (PR3 or elastase). A similar result was obtained in HUVEC (data not shown).
(TIF)

Figure S3 **Detecting internalization of PAR1 induced by either PR3 or elastase.** Internalization of PAR1 of surface of adherent HUVEC was inhibited by temperature (performed on ice, 4°C), or by pre-incubated cells in Medium 199 containing a specific inhibitor, Dynasore (50 µM). All experimental conditions contained 0.02% DMSO (the vehicle for Dynasore). HUVEC were then treated with 1 or 5 µg/ml PR3 (34.5–172 nM) or 1 or 5 µg/ml elastase (33.9–169.5 nM) or 10 U/ml thrombin. The extent to which PR3 and elastase removed surface PAR1 by internalization was detected using a WEDE antibody under these inhibitory conditions. Fig. S3A shows data from cells maintained on ice throughout the experiment. Fig. S3B compares the effect of temperature/chemical inhibition. The statistical symbols indicated significant difference between *protease treatment compared to the non-protease treated control or between +protease treatment in the presence of an inhibitor of internalization i.e. either cold (4°C) or Dynasore (50 µM) compared to protease treatment at 37°C in the absence of an inhibitor.
(TIF)

Figure S4 **Antibody binding sites in the PAR1 and PAR2 sequences, and PR3-or elastase-induced modulation of receptor structure.** Fig. S4 firstly shows the binding sites of specific anti-PAR1 and anti-PAR2 antibodies and then illustrates non-activatory proteolysis by serine proteases leading to inactivation of PAR1 (disarming). There was no associated glomerular endothelial cell PAR2 activation or disarming (under the same conditions).
(TIF)
