# Supplementary figures and images for: PR3 and Elastase Alter PAR1 Signaling and Trigger vWF Release via a Calcium-Independent Mechanism from Glomerular Endothelial Cells
Source: PLoS One. 2012 Aug 29;7(8):e43916. doi: 10.1371/journal.pone.0043916 (PMC3430624; doi:10.1371/journal.pone.0043916)

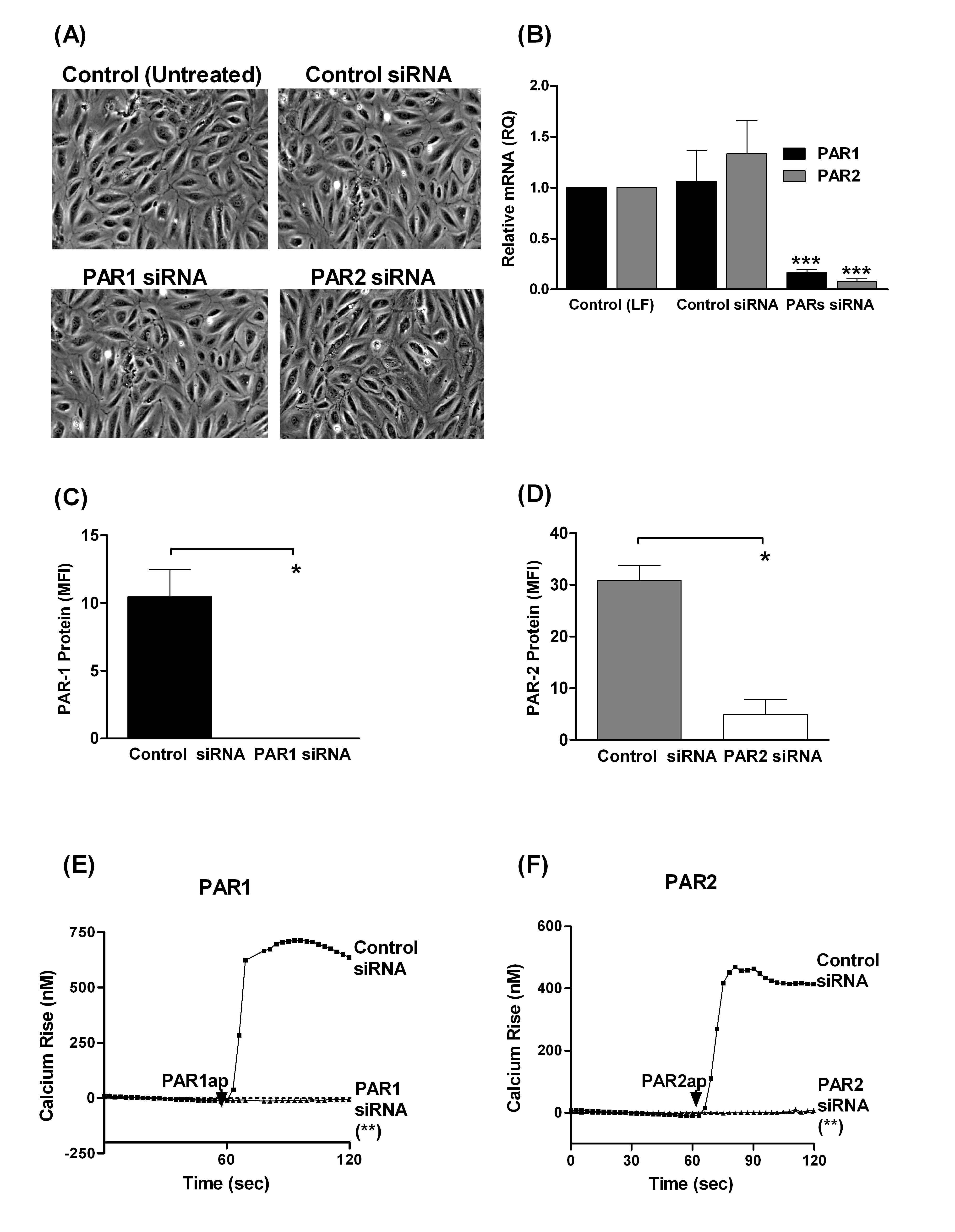

Supplement: Figure S1 — PAR1 and PAR2 mRNA and protein knockdown by siRNA. Fig. S1A shows phase contrast photomicrographs of (i) untreated HUVEC and cells exposed to (ii) non-targeting siRNA (Control siRNA) and (iii) PAR1 siRNA and (iv) PAR2 siRNA. PAR1 (black) and PAR2 (grey) of GEC mRNA levels were abolished by siRNA treatment. This silencing of PAR mRNA was detected, 48 hr after a 4 hr siRNA treatment. Data were expressed as relative expression compared to lipofectamine treated cells (Fig. S1B n = 5–6). The reduction in PAR1 (Fig. S1C n = 3) and PAR2 (Fig. S1D n = 3) protein levels induced by siRNA treatment was detected by flow cytometry using WEDE (anti-PAR1) and SAM11 (anti-PAR2) antibodies. The reduction in glomerular endothelial cell PAR1 (Fig. S1E) and PAR2 (Fig. S1F) calcium signal due to siRNA knockdown was also assessed. These are representative calcium traces (n = 3, for PAR1 p = 0.0493*, for PAR2 p = 0.0081**). (TIF) [file pone.0043916.s001.tif]

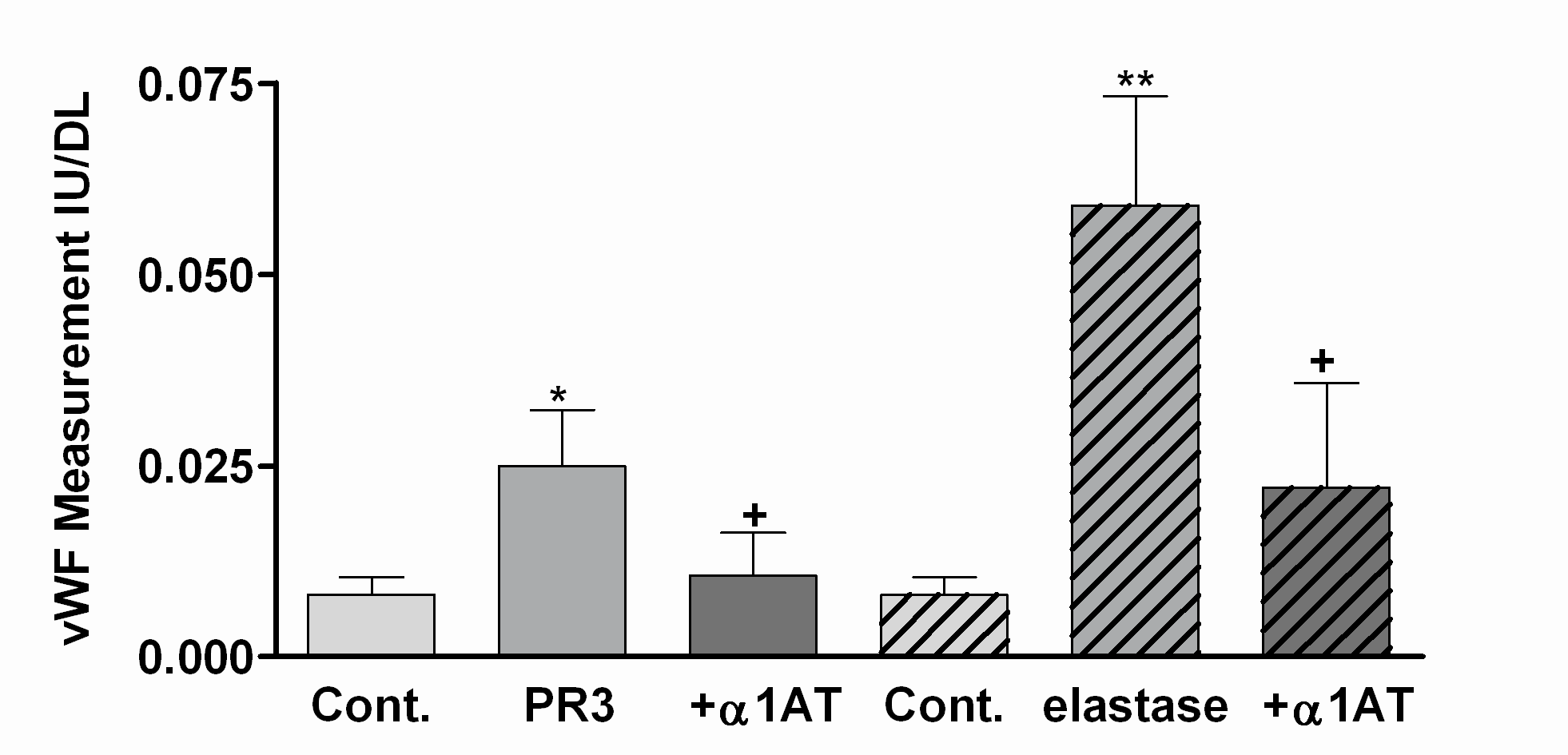

Supplement: Figure S2 — PR3 or elastase induced GEC vWF release in the presence of alpha anti-trypsin. Fig. S2 shows that GEC vWF release in response to PR3 or elastase. This is abolished in the presence of alpha anti-trypsin (+α1AT). The statistical symbols indicated significant difference either compared to *untreated controls (Cont.) or +compared to protease treatment only (PR3 or elastase). A similar result was obtained in HUVEC (data not shown). (TIF) [file pone.0043916.s002.tif]

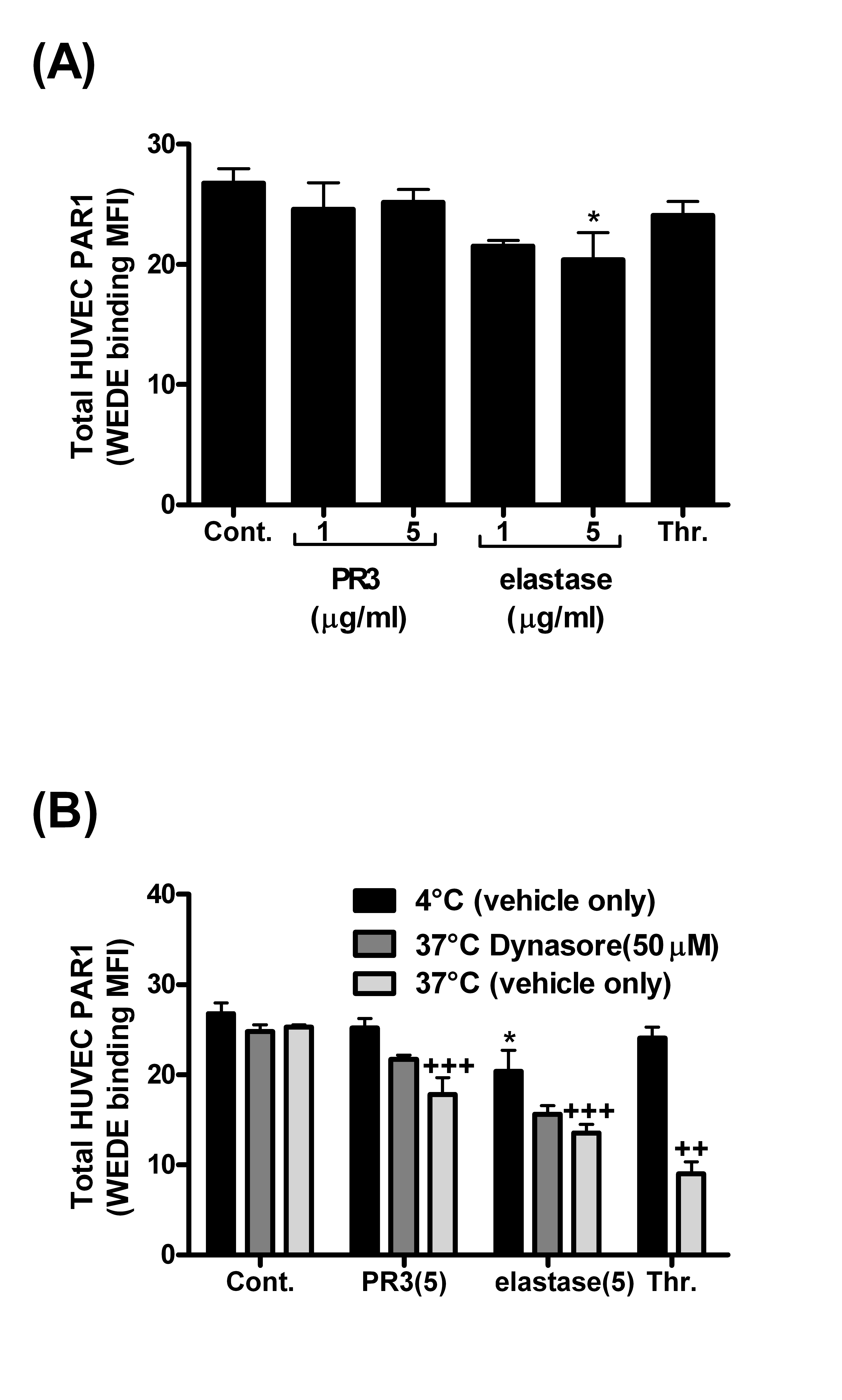

Supplement: Figure S3 — Detecting internalization of PAR1 induced by either PR3 or elastase. Internalization of PAR1 of surface of adherent HUVEC was inhibited by temperature (performed on ice, 4°C), or by pre-incubated cells in Medium 199 containing a specific inhibitor, Dynasore (50 µM). All experimental conditions contained 0.02% DMSO (the vehicle for Dynasore). HUVEC were then treated with 1 or 5 µg/ml PR3 (34.5–172 nM) or 1 or 5 µg/ml elastase (33.9–169.5 nM) or 10 U/ml thrombin. The extent to which PR3 and elastase removed surface PAR1 by internalization was detected using a WEDE antibody under these inhibitory conditions. Fig. S3A shows data from cells maintained on ice throughout the experiment. Fig. S3B compares the effect of temperature/chemical inhibition. The statistical symbols indicated significant difference between *protease treatment compared to the non-protease treated control or between +protease treatment in the presence of an inhibitor of internalization i.e. either cold (4°C) or Dynasore (50 µM) compared to protease treatment at 37°C in the absence of an inhibitor. (TIF) [file pone.0043916.s003.tif]

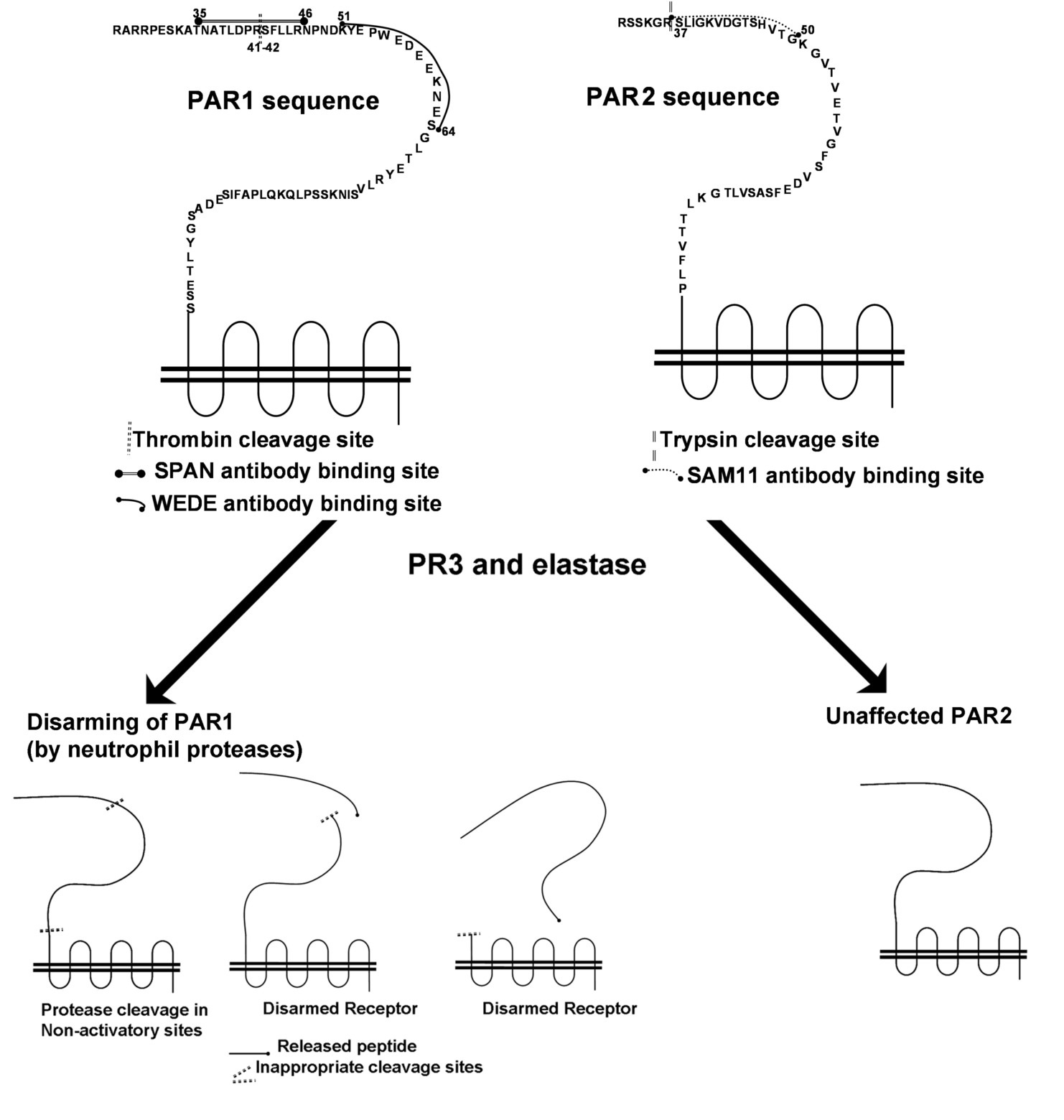

Supplement: Figure S4 — Antibody binding sites in the PAR1 and PAR2 sequences, and PR3-or elastase-induced modulation of receptor structure. Fig. S4 firstly shows the binding sites of specific anti-PAR1 and anti-PAR2 antibodies and then illustrates non-activatory proteolysis by serine proteases leading to inactivation of PAR1 (disarming). There was no associated glomerular endothelial cell PAR2 activation or disarming (under the same conditions). (TIF) [file pone.0043916.s004.tif]
